# The relation between kinematic synergy to stabilize the center of mass during walking and future fall risks: a 1-year longitudinal study

**DOI:** 10.1186/s12877-021-02192-z

**Published:** 2021-04-13

**Authors:** Momoko Yamagata, Hiroshige Tateuchi, Itsuroh Shimizu, Junya Saeki, Noriaki Ichihashi

**Affiliations:** 1 Graduate School of Human Development and Environment, Kobe University, 3-11 Tsurukabuto, Nada-ku, Kobe, Hyogo 657-0011 Japan; 2 Graduate School of Medicine, Kyoto University, 53 Kawahara-cho, Shogoin, Sakyo, Kyoto, 606-8507 Japan; 3 Research Fellow of the Japan Society for the Promotion of Science, 5-3-1 Kojimachi, Chiyodaku, Tokyo, 102-0083 Japan; 4 Fukui General Clinic, 1-42-1 Nittazuka, Fukui-shi, Fukui, 910-0067 Japan; 5 Faculty of Sport Sciences, Waseda University, 2-579-15 Mikajima, Tokorozawa, Saitama, 359-1192 Japan

**Keywords:** Falls, Aging, Center of mass, Segmental coordination, Uncontrolled manifold

## Abstract

**Background:**

Incorrect body weight shifting is a frequent cause of falls, and the control of the whole-body center of mass (CoM) by segmental coordination is essential during walking. Uncontrolled manifold (UCM) analysis is a method of examining the relation between variance in segmental coordination and CoM stability. However, no prospective cohort study has thoroughly investigated how variance in segmental configurations to stabilize the CoM relates to future falls. This study explored whether variance to stabilize the CoM was related to future falls.

**Methods:**

At the baseline visit, 30 community-dwelling older adults walked 20 times on a 6-m walkway. Using kinematic data collected during walking by a three-dimensional motion capture system, UCM analysis was performed to investigate how segmental configuration contributes to CoM stability in the frontal plane. One year after the baseline visit, we evaluated whether the subjects experienced falls. Twelve subjects had experienced falls, and 16 had not. Comparisons of variance between older adults with and without falls were conducted by covariate analysis.

**Results:**

No significant differences in variance were found in the mediolateral direction, whereas in the vertical direction, older adults with fall experiences had a greater variance, reflecting an unstable CoM, than those with no fall experiences.

**Conclusions:**

We verified that the high variance in segmental configurations that destabilize the CoM in the vertical direction was related to future falls. The variables of UCM analysis can be useful for evaluating fall risk.

## Background

Falls in older adults have several causes and lead to increased medical expenses and fatal injuries [1]. Given that most falls occur due to moving the center of mass (CoM) outside the base of support following tripping and slipping, a well-controlled CoM is essential for successful walking. In particular, the unstable postural control and falls in the frontal plane is a great risk of hip fracture, leading to low quality of life and permanent disability [2, 3].

The body segments move in a coordinated manner to control the CoM trajectory during walking, and synergistic control of the abundant body segments is necessary to succeed in the walking task [4]. The strength of synergy can be quantified using uncontrolled manifold (UCM) analysis [5]. The synergy calculated by UCM analysis is defined as a neural organization that ensures the coordination of the elements (i.e., body segments in this study) to stabilize the important performance variable in each task (i.e., CoM trajectory during walking in this study) [6]. Using UCM analysis, the segment variance across repetitive tasks is categorized into two types of variance: variance that reflects a stable performance variable (V_UCM_) and variance that reflects an unstable performance variable (V_ORT_). The synergy index computed from V_UCM_ and V_ORT_ is a measure to quantify the strength of synergy that contributes to the stability of the performance variable [6]. A high synergy index resulting from an increase in V_UCM_ or decrease in V_ORT_ reflects a strong synergy, implying that an abundant set of elements, i.e., body segments, work together in a coordinated manner to control task-specific performance variables, i.e., CoM trajectories.

Previously, we explored the relationship between falls and UCM indices i.e., V_UCM_, V_ORT_, and synergy index, using the swing foot position as a performance variable [7]. We found that older adults with a fall history exhibited a higher synergy index than older adults with no fall history, implying that older adults with a fall history use high segment coordination to maintain the stability of swing foot trajectories. Similar results were observed for stroke patients and older adults with a high risk of future falls [8, 9]. Such a person, however, is not a “good walker” and is very unlikely to have higher walking stability than a healthy person. While control of the swing foot is needed ﻿as an end-effector of a multiple-degree-of-freedom system, once the CoM becomes unstable, the body segments, including lower limb segments, have to be adjusted to maintain posture balance. Thus, the preferential control of swing foot and the excessive-high segmental configurations to stabilize swing foot during walking might lead to the failure of CoM control instead, that is, a decrease in synergy index stabilizing CoM position [10]. However, no study has investigated the relationship between fall experiences and UCM indices to control CoM trajectories.

The purpose of this prospective cohort study was to determine whether UCM indices that reflect the stable CoM trajectory in the frontal plane are related to future fall risk. Our hypothesis was that the low segment flexibility pattern – that is, high V_ORT_, low V_UCM_, and low synergy index – at the baseline visit is related to a high risk of falling in the future.

## Methods

### Subjects

Community-dwelling older adults who were at least 60 years old were recruited via flyers. Thirty volunteers participated, and they gave written informed consent according to the procedure approved by the Research Ethics Committee of Kyoto University. The inclusion criteria, which were evaluated via interview, were as follows: a person without neurological disorders or musculoskeletal injuries and a person who could walk without assistance. In this prospective study, we used the same cohort as in our previous study [9].

### Experiment at baseline visit

At the baseline visit, the subjects walked from a marked starting point to an ending point on a 6-m walkway at a comfortable speed 20 times. A 3–5 min rest was provided after every 5 trials to minimize fatigue.

**Fig. 1:**
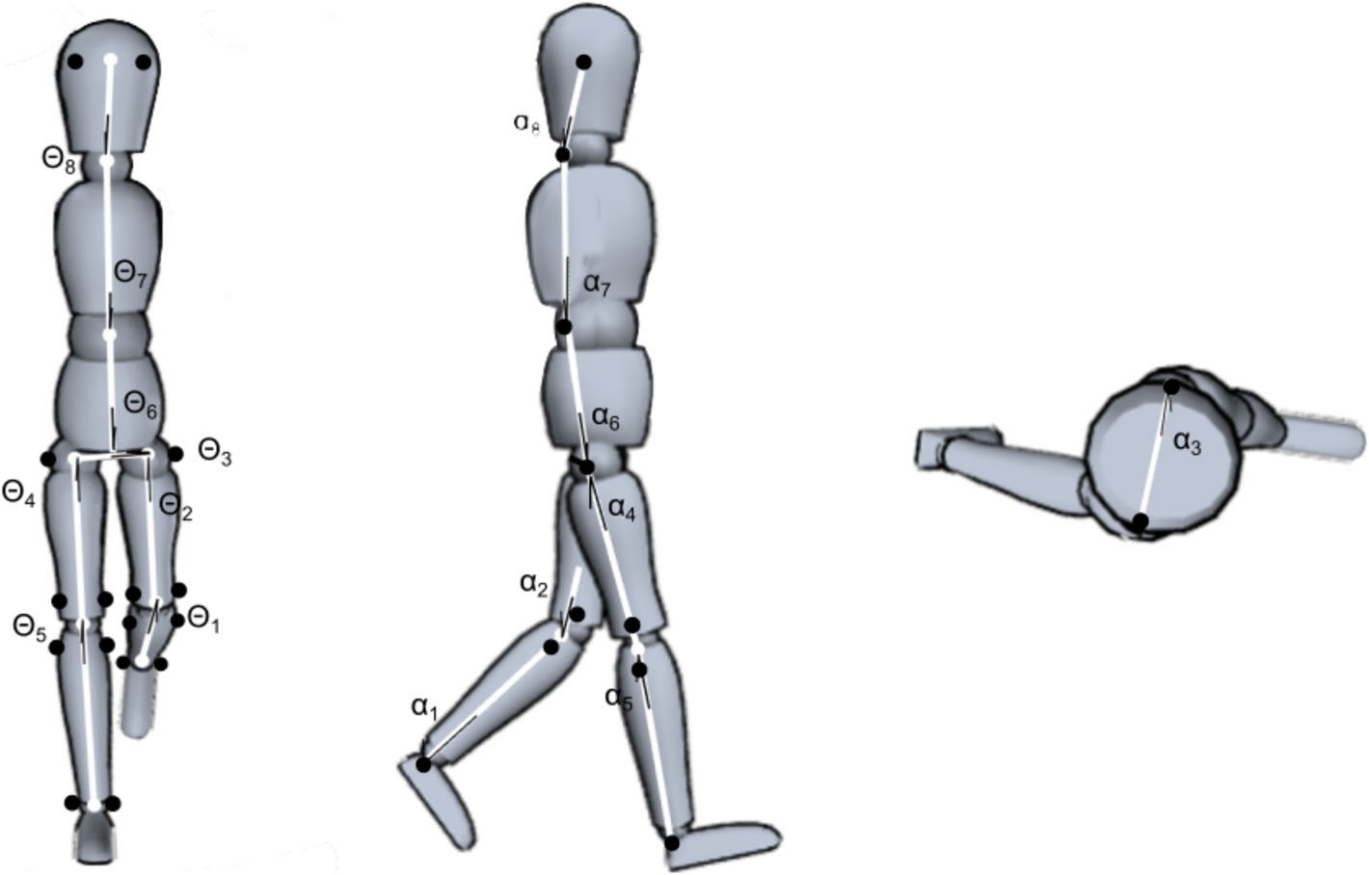
Illustrations of the segmental angles for the geometric model. Eight segments and 16 degrees of freedom are used for the analysis; 8 degrees of freedom in the frontal plane (Θ_1_: left shank, Θ_2_: left thigh, Θ_3_: pelvis, Θ_4_: right thigh, Θ_5_: right shank, Θ_6_: lower trunk, Θ_7_: upper trunk, Θ_8_: head) and 8 degrees of freedom in sagittal and transverse plane (α_1_: left shank, α_2_: left thigh, α_3_: pelvis, α_4_: right thigh, α_5_: right shank, α_6_: lower trunk, α_7_: upper trunk, α_8_: head). The segments were defined from marker data (black circle) as follows: shank; ankle to knee joint, thigh; knee to hip joint, pelvis; left hip to right hip joint, lower trunk; middle point of both hip joints to T10, upper trunk; T10 to C7, neck; C7 to middle point of foreheads

To define the body segment (Fig. 1), a physical therapist with 6 years of experience placed reflective markers on the 7th cervical vertebra (C7) and 10th thoracic vertebra (T10) and on both sides of each subject at the following locations: forehead, greater trochanter, medial and lateral femoral condyles, medial tibia condyle, head of fibula, medial condyle of tibia, medial and lateral malleolus. Kinematic data were collected with eight infrared cameras (VICON MX; Vicon Motion Systems, Oxford) at 100 Hz. We defined the mediolateral direction relative to the global laboratory coordinate system and the frontal plane perpendicular to the global anterior-posterior axis, corresponding to walking orientation.

The data of the single support (SS) phase from toe-off until initial contact in the dominant foot were normalized by time (0–100%). To include steps during steady-state walking excluding acceleration and deceleration phases, two steps in the dominant foot after excluding the first four steps, i.e., a total of 40 steady steps, were used for further analysis [11]. Forty steps were chosen as a sufficient repetitive number for a reasonable approximation of UCM indices [12, 13]. Each joint center was calculated from marker data, and the CoM was defined as the position of the sum of each segmental center of mass, i.e., both shanks, both thighs, pelvis, lower trunk, upper trunk, and head [7, 14, 15]. We calculated the average and variability of the CoM position across 40 steps. The variability was calculated as the standard deviation. The fall efficacy scale (FES) was evaluated as physical information at the baseline visit [16]. The FES is a reliable and valid instrument to assess fear of falling, and the FES score is related to walking ability in older adults [16].

### Future falls

One year after the baseline visit, we sent a questionnaire to all subjects asking, “Have you experienced falls within a year?” [17, 18]. When the subjects had experienced falls, we also asked whether they were injured when falling. The subjects were divided into two groups (older adults with fall experiences: Fallers; older adults without fall experiences: Nonfallers). Further data analysis was conducted on the subjects who answered all questions; in this study, the data of two subjects were excluded because of a lack of responses.

### UCM analysis

Since body segments work in a coordinated manner to control the CoM position during walking, UCM analysis ﻿of multi-segment coordination is a feasible and valid method [5, 19].

CoM trajectories in the mediolateral (CoM_ML_) and vertical (CoM_V_) directions were used as performance variables as follows [6, 14]:

$$ CoM={x}_0+\sum \limits_{i=1}^8{M}_i{x}_i $$

where:

$$ {x}_i=\sum \limits_{j=1}^i{C}_j{L}_i\cos {\alpha}_j\sin {\theta}_j\ \mathrm{for}\ {CoM}_{ML} $$

$$ {x}_i=\sum \limits_{j=1}^i{C}_j{L}_i\cos {\alpha}_j\cos {\theta}_j\ \mathrm{for}\ {CoM}_V $$

and:

$$ {C}_j=1\ \mathrm{for}\ j<i $$

where *x*_*0*_ and *z*_*0*_ are the segmental positions of the absolute coordinate system in the ML and V directions, *Θ*_*1*_*,..., Θ*_*8*_ are the defined segmental angles in the frontal plane, *α*_*1*_*,..., α*_*8*_ are the defined segmental angles in the sagittal and transverse planes, *C*_*1*_*,..., C*_*8*_ are the estimated locations of the segmental center of mass, *M*_*1*_*,..., M*_*8*_ are segmental masses normalized by total body mass, and *L*_*1*_*,..., L*_*8*_ are the lengths of the segments [14, 15].

A Jacobian system (*J*) was used to link the changes in elemental variables (segmental angles in 16 DoFs) and changes in the performance variable (CoM trajectory). *J* is the matrix of partial derivatives of changes in the CoM trajectory with respect to segmental angles, and the null space (ε) is the (*n* − *d*) vector represented by the dimensions in the segmental configuration space (*n* = 16) and CoM trajectory (*d* = 1). At every portion of the SS phase, the differences between the segmental configurations and their mean $\Big(\uptheta -\overline{\theta \Big)}$ were projected onto the null space:

$$ {\theta}_{UCM}=\sum \limits_{i=1}^{n-d}\left(\theta -\overline{\theta}\right)\ast {\varepsilon}_i $$

and the space orthogonal to the null space:

$$ {\theta}_{ORT}=\left(\theta -\overline{\theta}\right)-{\theta}_{UCM} $$

The variance in the segment configuration that does not affect the CoM_ML_ or CoM_V_ (*V*_*UCM*_) was calculated as the average of the squared length of *θ*_*UCM*_ across 40 steps, and normalized by the DoFs within the UCM subspace:

$$ {V}_{UCM}={\left(n-d\right)}^{-1}\ast {N}^{-1}\ast \sum {\left({\theta}_{UCM}\right)}^2 $$

The variance in the segment configuration that affects the CoM_ML_ or CoM_V_ (*V*_*ORT*_) was calculated as the average of the squared length of *θ*_*ORT*_ across 40 steps, and normalized by the DoFs within the orthogonal subspace:

$$ {V}_{ORT}={d}^{-1}\ast {N}^{-1}\ast \sum {\left({\theta}_{ORT}\right)}^2 $$

∆V was computed from *V*_*UCM*_ and *V*_*ORT*_ as below:

$$ \Delta  \mathrm{V}=\frac{V_{UCM}-{V}_{ORT}}{V_{TOT}}, $$

where

$$ {V}_{TOT}=\left(\frac{1}{n}\right)\left(d{V}_{ORT}+\left(n-d\right){V}_{UCM}\right). $$

For a normal distribution, Fisher’s *z*-transformation was applied to ∆V according to previous studies (∆*V*_*Z*_) [20].

SS includes large alterations in kinematics and kinetics [21], and changes in the inverted U-shaped curve of ∆V_Z_ were expected [7, 8]; thus, the SS phase was divided into the first 1/3 (Early SS), second 1/3 (Mid SS), and third 1/3 (Late SS). For further analysis, the average UCM indices during each phase were calculated.

### Statistical analysis

To test the effects of CoM position and CoM variability during the three SS phases on future falls, two-way ANOVAs (*Phase*: Early-, Mid-, Late-SS phases, and *Group*: Faller and Nonfaller) were performed.

To test the effects of *Phase* (Early-, Mid-, and Late-SS phase) and *Group* (Faller and Nonfaller) on UCM indices, multivariate analysis of covariance (MANCOVA) adjusted for walking speed was performed in the ML and V directions separately. The walking speed was used for adjustment based on a previous study that revealed the effects of walking speed on UCM indices [8, 22]. When significant major effects or interactions were detected, we performed post hoc comparisons. All analyses were performed with SPSS (Version 18, PASW Statistics, Chicago), and the significance level was set at *p* = 0.05.

As secondary tests, we calculated Spearman’s correlation coefficient to explore the relations between UCM indices for CoM trajectory and two factors: UCM indices for swing foot trajectory calculated using a previous geometric model [7]; and the FES score for the fear of falling. The relations between UCM indices for the CoM trajectory and swing foot trajectory reveal whether the preferential control of swing foot leads to the failure of CoM control instead. Considering that fear of falling changes gait patterns such as stiffening strategy [23], the relations of UCM indices for the CoM trajectory with the FES score would also provide important implications to develop a deeper understanding of falling mechanisms.

Before statistical analysis, the data were tested for statistical assumptions of normality and sphericity. For nonnormal or nonspherical data, we used log-transformation or the Greenhouse-Geisser correction, respectively.

## Results

**Fig. 2:**
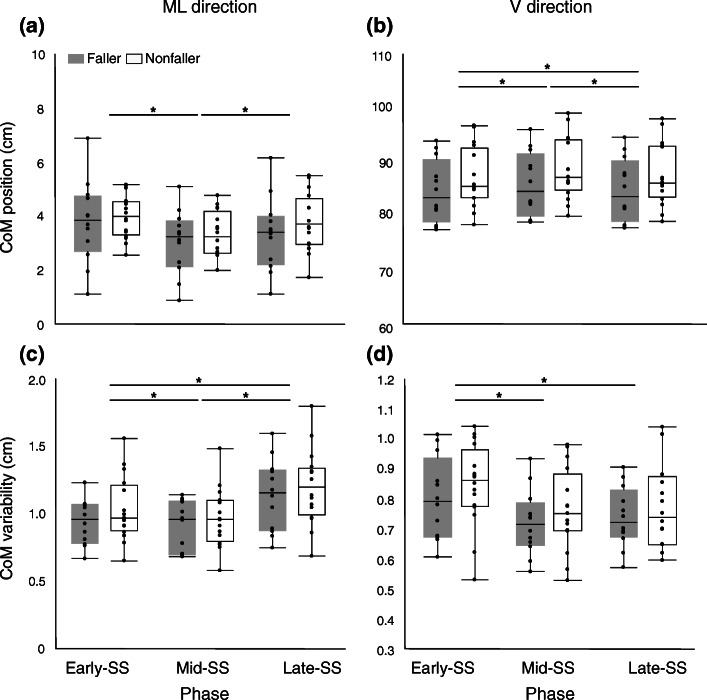
Box plots of the CoM position (**a, b**) and CoM variability (**c, d**). Box plots of CoM indices in Faller (grey boxes) and Nonfaller (white boxes) for three phases, Early-, Mid-, and Late-SS phases are shown. Dots represent values in each subject. The horizontal line displays the median and the box-edges display the 25th and 75th percentiles. Upper panels: CoM position in the ML (**a**) and in V (**b**) directions; Lower panels: CoM variability in the ML (**c**) and V (**d**) directions. V direction: vertical direction, ML direction: mediolateral direction, SS: single stance phase. ﻿Statistically significant major effects are shown with one star (*p* < 0.05)

Two subjects who provided no responses on the questionnaire were excluded from the analyses. The subjects were divided into Faller (*n* = 12) and Nonfaller (*n* = 16) groups. Physical characteristics are shown in Table 1. Walking speed was lower in Fallers than in Nonfallers (*p* = 0.033), but the other characteristics of Fallers were similar to those of Nonfallers. CoM_ML_ displacements during Early- and Late-SS phases were significantly larger than those during the Mid-SS phase (*Phase* effect: F (2,52) = 24.7; *p* < 0.001, Fig. 2a). CoM_V_ displacements were greater in the Mid-SS phase than in the Late-SS phase and greater in the Late-SS phase than in the Early-SS phase (*Phase* effect: F (2,52) = 83.8; *p* < 0.001, Fig. 2b). CoM_ML_ variability was greater in the Late-SS phase than in the Early-SS phase and greater in the Early-SS phase than in the Mid-SS phase (*Phase* effect: F (2,52) = 85.5; *p* < 0.001, Fig. 2c). CoM_V_ variability was significantly greater in the Early-SS phase than in the Late-SS and Mid-SS phases (*Phase* effect: F (2,52) = 26.1; *p* < 0.001, Fig. 2d). No *Group* effect or interaction was found in CoM displacements and variabilities.

**Table 1:** Physical characteristics

	Nonfaller (n = 16)	Faller (*n* = 12)
Age (years)	73.8 ± 7.9	78.0 ± 4.7
Height (m)	1.6 ± 0.08	1.54 ± 0.11
Weight (kg)	58.4 ± 8.3	52.2 ± 8.1
BMI	22.7 ± 2.5	21.9 ± 2.2
Gait Velocity (m/s) *	1.3 ± 0.1	1.1 ± 0.1
FES score	34.9 ± 4.4	33.3 ± 4.5

*p < = 0.05 between Nonfaller and Faller

**Fig. 3:**
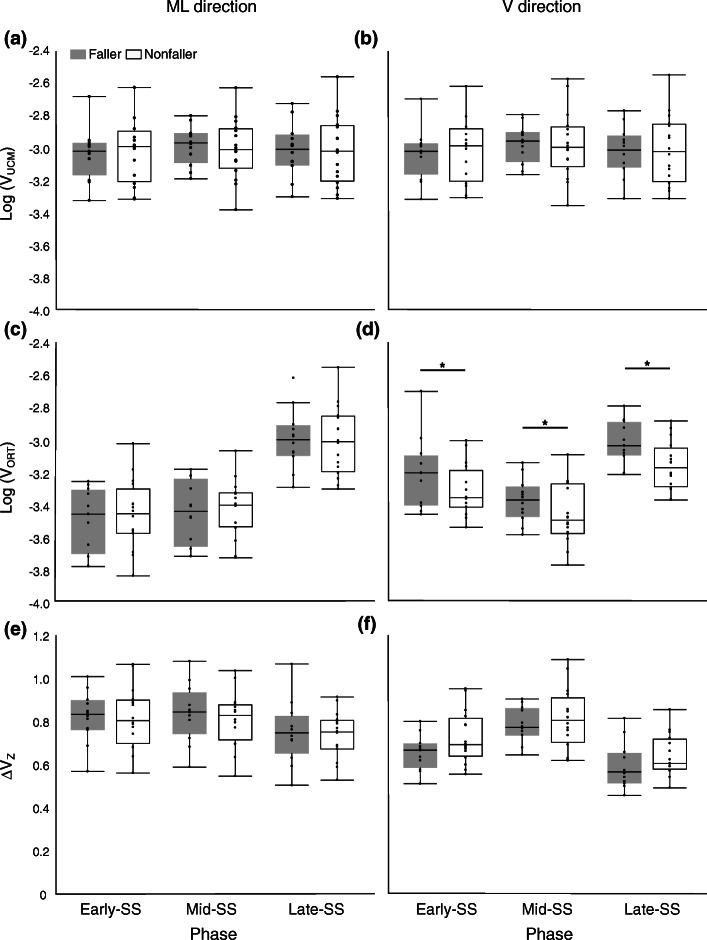
Box plots of the variance components. Box plots of CoM indices in Faller (grey boxes) and Nonfaller (white boxes) for three phases, Early-, Mid-, and Late-SS phases are shown. Dots represent values in each subject. The horizontal line displays the median and the box-edges display the 25th and 75th percentiles. Upper panels: V_UCM_ in the ML (**a**) and V (**b**) directions, Middle panels: V_ORT_ in the ML (**c**) and V (**d**) directions, Lower panels: ΔV_Z_ in the ML (**e**) and V (**f**) directions. Statistically significant major effects are shown with one star (*p* < 0.05). For abbreviation, see the caption for Fig. 2

Figure 3 shows the average UCM indices during each phase. *Phase* effects were seen in V_ORT_ and ΔV_Z_ in the V direction (V_ORT_: F (2,50) = 51.56; *p* < 0.001, ΔV_Z_: F (2,50) = 3.41; *p* < 0.001). V_ORT_ was significantly greater in the Late-SS than in the Early-SS phase and was greater in the Early-SS phase than in the Mid-SS phase, whereas ΔV_Z_ was significantly greater in the Mid-SS phase than in the Early-SS phase and greater in the Early-SS phase than in the Late-SS phase. A *group* effect was seen in the V_ORT_ in the V direction (F (1,25) = 5.44; *p* = 0.028); Fallers displayed a significantly greater value than Nonfallers. No major effects in other indices or interactions were found for any indices.

**Fig. 4:**
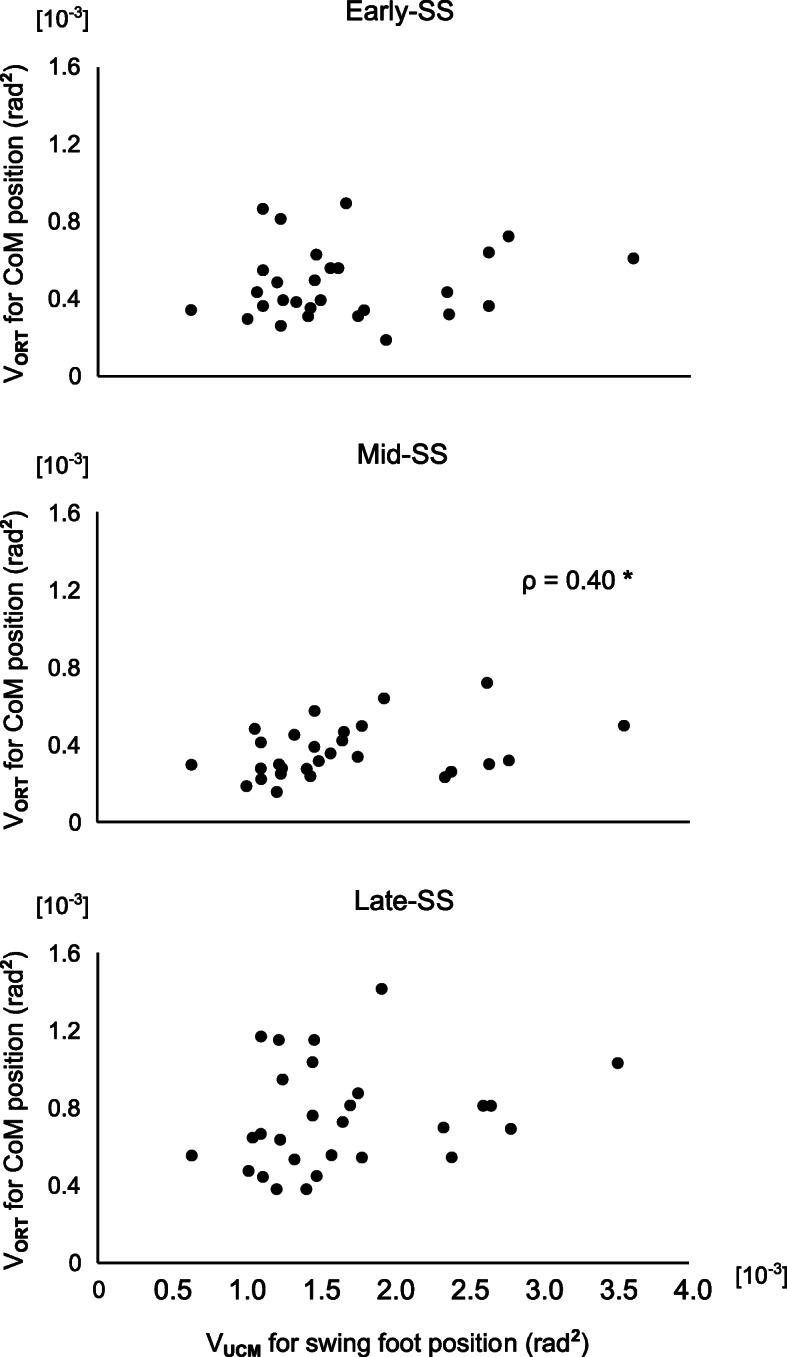
The correlations between V_ORT_ for the vertical CoM position and V_UCM_ for the vertical swing foot position. The horizontal axes represent V_UCM_ for swing foot position, and the vertical axes represent V_ORT_ for CoM position. Upper panels: the correlations in the Early-SS phase, Middle panels: the correlations in the Mid-SS phase, Lower panels: the correlations in the Late-SS phase. Statistically, significant correlations are shown with one star (*p* < 0.05). For abbreviation, see the caption for Fig. 2

Regarding relations between UCM indices for the CoM trajectory and the other factors, in the Mid SS phase, there was a significant correlation between V_ORT_ for vertical CoM trajectory and V_UCM_ for vertical swing foot trajectory (ρ = 0.40, *p* = 0.03; Fig. 4), indicating that V_ORT_ for the vertical CoM trajectory increased with an increase in V_UCM_ for vertical swing foot trajectory. No correlations between UCM indices and FES scores were found.

## Discussion

The purpose of this study was to investigate whether UCM indices to control the CoM trajectory during walking were related to future falls. The CoM displacement and variability were not significantly different between Fallers and Nonfallers in the ML and V directions. No significant effect of fall experiences was found for UCM indices in the ML direction, and our hypothesis that high V_ORT_, low V_UCM_, and low synergy index at the baseline visit are related to a high risk of falling in the future was not supported. In the V direction, the V_ORT_ in Fallers was greater than that in Nonfallers, with no difference in V_UCM_ and ΔV_Z_. Thus, our hypothesis was partly supported in the V direction.

Previous studies showed that patients with Down syndrome and stroke patients displayed greater V_UCM_ to stabilize the CoM than healthy people, in contrast to our findings [5, 19]. Motor and sensory dysfunctions would occur in such patients due to their diseases, and to compensate for the disorders, they might establish a different strategy during walking, such as an increase in V_UCM_ to maintain CoM stability. On the other hand, because motor disorders gradually occur with aging, older adults who will experience falls would not be able to use the same compensatory strategy used by patients who suddenly experience the disorders, resulting in an increase in V_ORT_ and fall experiences.

The V_ORT_ in the V direction was significantly greater in Fallers than in Nonfallers, and the difference was relatively large (effect size f: 0.43). During walking, forward CoM movement while maintaining posture stability against the change in ground reaction force is necessary, especially during the SS phase, when posture is readily rendered unstable [21]. Previous studies have shown that high muscle coactivation and low segment coordination result in low posture stability due to high signal-dependent noise and the transmission of perturbations along the vertical body axis [24, 25]. The increased vertical perturbation by external forces, such as ground reaction force, possibly resulted in high V_ORT_ that reflects an unstable CoM and high risk of future falls. The development of proper therapies leading to a decrease in V_ORT_ is needed for future study.

Despite falls often occurring by incorrect transfer of the CoM to outside the base of support, interestingly, only V_ORT_ in the V direction constituted an adequate index to identify Fallers and Nonfallers. Earlier studies showed a directional difference in the aspects of locomotor control; less able subjects had high variability in the V direction due to low variability in the ML direction (i.e., Bernsteinian freezing of degrees of freedom) [26, 27]. Fallers in this study, however, would not use such a walking strategy since ΔV_Z_ reflecting freezing gait was similar to Nonfallers. To develop a deeper understanding of a walking pattern that leads to high V_ORT_ and future falls, evaluating the segment configurations serving to stabilize anterior-posterior CoM might be needed.

Our previous study observed that older adults with a fall history displayed a highly stable vertical swing foot through the segmental configuration during walking [7], and similar findings were seen in patients with motor disability [10]. In addition to these reports, the current study revealed that a high V_UCM_ for vertical swing foot trajectory was related to a high V_ORT_ for vertical CoM trajectory in the Mid-SS phase, implying that older adults with well-controlled swing foot trajectory during walking displayed the loss of vertical CoM control instead. While high elevations of swing foot in the Mid-SS phase in older adults are used as one of the conservative walking patterns to prevent trip-related falls [28, 29], in the clinical field, it is necessary to keep in mind the possibility that the excessive control of vertical swing foot trajectory through the segmental configurations may lead to the loss of the control of vertical CoM trajectory, and those walking patterns might lead to falls.

CoM-related variables, walking speed, and the FES have previously been used as indices for fall risks [30–32]. However, there were no differences in CoM displacement, CoM variability, or FES scores between groups. As we found a lower walking speed in Fallers, the V_ORT_ in Fallers was greater than that in Nonfallers after adjusting for walking speed. The variance in segmental configurations that reflects CoM instability can serve as an index to evaluate potential fall risks independently of walking speed even for subjects with a normal functional level on indices of fall risks, such as CoM-related variables and FES.

There were some limitations in this study. First, a questionnaire was applied to distinguish Fallers and Nonfallers, which might have led to recall bias. Despite the limitations, we believe this common method showed relatively low recall bias, as it occurs over a period of 1 year. Second, there was no information about the causes of falls, although the required performance variable might be different among people with different falling risks. Third, 6 m-walkway might be short to evaluate the steady-state walking. Longer walkway would be better to include sufficiently steady steps for reasonable UCM indices. Additionally, Type II error might have occurred because of the small sample size. For example, the mean age of Fallers was 4 years older than that of Nonfallers; although this difference was not significant, our findings may have been affected by age. Given that CoM movements during walking would change with aging [32], greater V_ORT_ in Fallers than in Nonfallers might also be caused by aging. Finally, selection bias might have affected the results, given the high rate of falls in this study.

Other methodological considerations include the geometric model we used in this study. We did not include segments of the upper extremities. Since arm swing is one of the important components for controlling the CoM trajectories during walking and arm swing would change the CoM trajectories [33], a modified model considering the movements of upper extremities is needed in future research. Additionally, we did not focus on the gait in anteroposterior direction. Given major causes of falls such as tripping and slipping will increase the CoM movements in the sagittal plane [34], research on the relationships between sagittal CoM trajectories and segment coordination would reveal important implications for future falls.

## Conclusions

Overall, our study is the first to demonstrate the relationships between kinematic synergy to stabilize the CoM during walking and future fall risks. Although some measures that have been previously used to evaluate fall risk (e.g., CoM variability and FES scores) could not distinguish between older adults with and without future falls, the high variance in segmental configurations that affects CoM stability in the vertical direction was related to future falls independent of walking speed. The UCM index can predict future falls and serve as an index for fall risks even in subjects with a normal level on some previous fall risk indices.

## Data Availability

All datasets in this study are available from the corresponding author on reasonable request.

## Abbreviations

CoM: Center of mass
BoS: Base of support
SS phase: Single support phase
UCM: Uncontrolled manifold
V_UCM_: Variance in the UCM subspace
V_ORT_: Variance in the subspace orthogonal to the UCM
C7: 7th cervical vertebra
T10: 10th thoracic vertebra
ML direction: Mediolateral direction
V direction: Vertical direction
